# Heuristic Resource Reservation Policies for Public Clouds in the IoT Era

**DOI:** 10.3390/s22239034

**Published:** 2022-11-22

**Authors:** Omer Melih Gul

**Affiliations:** 1 Department of Electrical and Electronics Engineering, Middle East Technical University (METU), Ankara 06800, Turkey; omgul@metu.edu.tr or ogul@uottawa.ca; 2 School of Electrical Engineering and Computer Science, University of Ottawa, Ottawa, ON K1N 6N5, Canada

**Keywords:** public cloud, cost optimization, resource reservation, cloud computing

## Abstract

With the advances in the IoT era, the number of wireless sensor devices has been growing rapidly. This increasing number gives rise to more complex networks where more complex tasks can be executed by utilizing more computational resources from the public clouds. Cloud service providers use various pricing models for their offered services. Some models are appropriate for the cloud service user’s short-term requirements whereas the other models are appropriate for the long-term requirements of cloud service users. Reservation-based price models are suitable for long-term requirements of cloud service users. We used the pricing schemes with spot and reserved instances. Reserved instances support a hybrid cost model with fixed reservation costs that vary with contract duration and an hourly usage charge which is lower than the charge of the spot instances. Optimizing resources to be reserved requires sufficient research effort. Recent algorithms proposed for this problem are generally based on integer programming problems, so they do not have polynomial time complexity. In this work, heuristic-based polynomial time policies are proposed for this problem. It is exhibited that the cost for the cloud service user which uses our approach is comparable to optimal solutions, i.e., it is near-optimal.

## 1. Introduction

Fifth-generation (5G) communications technology is finally here with its promised low-latency performance and high speed. Many intriguing cloud computing developments loom with it. In addition, via the redefinition of business networks, 5G will change many roles of networks and cloud computing in storing, moving, and accessing data as innovation drives and provides digital business transformation many technological applications. The impact of 5G on cloud computing can be observed in transforming edge computing, redefining the function of the cloud, converging in the cloud, and the dawn of network cloudification [1].

Recently, wireless sensor networks (WSNs) have been applied to various fields, such as environment monitoring, manufacturing, critical infrastructure monitoring, healthcare, public safety systems, and military monitoring. On the other hand, as WSNs have limits of scalability, communication, computational power, memory, and energy, it is very important to manage the large number of WSN data efficiently. They need a scalable high-performance computing and massive storage infrastructure to process data in real time and store them, in addition to analyzing the processed information within its context, using inherently complex models to extract events of interest. As a promising technology to mitigate the limitations of WSNs, cloud computing provides a low-cost, scalable, virtualized solution for a flexible stack of software services, storage, and massive computing. Consequently, the sensor-cloud infrastructure has recently become popular, which provides a flexible, open, and reconfigurable platform for WSNs to shift their storage and computations to remote clouds in many controlling and monitoring applications [2]. To date, many studies have handled various integration concerns of WSNs with the cloud. Sensors can reach resources in the public clouds by resource reservation with a price depending on various price schemes provided by cloud service providers. The resource provisioning for sensors in public clouds still remains an open issue.

In the following subsection, we give the motivation of the paper and why resource reservation policies are important for WSNs in the 5G and Internet of things (IoT) era where sensors may need more computational resources provided by public clouds in the beyond 5G and IoT era. In the following, we present the common pricing schemes provided by cloud service providers. Then, we present the main contributions of the paper and outline the rest of the paper.

### 1.1. Motivation

With the advances in the beyond 5G and IoT era, more and more wireless-sensor-equipped devices are expected to be connected to the Internet for achieving connectivity through the world. These devices, especially the mobile ones such as ground nodes and uncrewed aerial vehicles (UAV), need computational resources to achieve their tasks while keeping their security and privacy. For example, a recent article [3] tackle energy-aware and quality-aware data collection problem where a UAV plans a trajectory to collect data from ground nodes. As the trajectory optimization problem tackled in these papers is harder than orienteering problem [4], which is a combination of two NP-hard problems (traveling salesman problem [5] and knapsack problem [6]), the computational workload is excessive for a UAV depending on the topology of the network. In this case, the UAV can benefit from the computational resources provided by the public cloud. On the other hand, ground robots in a robotics and wireless sensor network, especially cluster head robots, may need more computational resources depending on the workload of data fusion and the number of robots in their cluster. The book chapter [7] investigates blockchain-aided IoT platforms which monitor transportation and sense vehicles. This chapter also investigates the use of blockchain in robotic networks. The paper [8] investigates the use of blockchain in the Internet of drones. Depending on the required security level in these mobile systems, more complex blockchain protocols need to be applied, which brings the necessity for more computational resources in those systems. From another perspective, the cyberphysical systems which require RF-domain security solutions may need computational resources if they use deep learning techniques, as explained in the paper [9,10,11]. Hence, future cyberphysical systems and IoT devices will need more cloud-based computational resources.

Cloud providers are working intensively to build services, tools and infrastructures, whereas many mobile operators deploy 5G access networks to provide their customer best service. To improve the service experience for customers, public cloud service providers and 5G network operators can work together in several areas in the forthcoming years. Some can be listed as back-office systems, 5G mobile edge, private mobile networks, and network functions [12].

Resource provisioning has emerged as a promising technique which allocates virtualized resources to users. If cloud service providers accept the users’ requests for resources, they use resource-provisioning techniques for creating and allocating an appropriate number of VMs based on demand [13]. Furthermore, their main responsibility is ensuring users’ QoS-based needs fulfillment of service-level agreement (SLA) negotiations in addition to mapping incoming workloads/applications to resources [14]. Resource provisioning brings several advantages including reducing the makespan and response times for submitted workloads, reducing overprovisioning and underprovisioning, reducing the startup delay of VMs, providing fault tolerance capabilities, and reducing power consumption [15].

In the last decade, practices for dedicated access to computers belonging to users (individuals, organizations, etc.) have been replaced by those of on-demand access to resources shared among many users. Cloud computing enables significantly this shift by providing a pervasive and on-demand network access for shared regulatable computing resource [16].

Current studies consider that cloud service users (CSUs) demand resources from cloud service providers (CSPs) and CSPs allocate virtualized resources to CSUs by considering the needs of CSUs. During these requests, CSUs are faced with a big challenge because of the resource pricing schemes offered by CSPs. Resources are accessible on a spot (or on demand) and reservation basis. Resource reservation is done with a constant pricing scheme for a fixed contract duration. On the other hand, a reservation for a longer duration or more resources than the ones needed to cover the demands for resources cause a higher cost for CSUs and overprovisioning [17]. Nevertheless, if the resources are allocated only on a spot basis (no reservation is made in this case), then the cost for CSUs will again be high, because spot prices are generally more than reserved prices in general. From the CSUs’ perspective, to decrease the cost of total resource usage, efficient and low-complexity policies are required.

In this work, we tackle a resource provisioning problem occurring in public clouds, where we determine the quantity of resources to reserve for minimizing the costs of executing an application. For this purpose, we tackle the problem under multiple pricing schemes given in the following subsection.

### 1.2. Pricing Schemes

CSPs offer computing resources as a utility and software as a service (SaaS) over networks. CSUs pay for these services or resources depending on their usage. To optimize the cost of services and resources from a CSU’s perspective is quite a hard problem since the CSPs generally present nonfixed pricing models for utilizing their resources. For this purpose, we need to understand the common pricing models well.

Descriptions of common pricing models that CSPs offer are provided as follows.

- *Fixed cost*—CSPs charge resource instances according to their types and duration in terms of months or years. Here, for each fixed time duration, one price is assigned. The cost is found by multiplying that price with the number of service units or resource instances which users request. CSUs pay a fixed cost even if resources are never utilized the whole time.
- *Variable cost*—CSPs provide services to the users on a variable-cost pay-as-you-go basis determined by their volume of transactions and the number of users. CSPs charge resource instances according to their types and usage (e.g., per hour) and with no long-term commitments or upfront payments. Resources are allocated on an on-demand basis which means that a user does not need to make a payment unless a resource is used.
- *Hybrid cost*—a mixture of fixed and variable costs, which includes both variable and fixed parts.
- *Flexible cost*—Resource instances are charged by the CSPs according to their time and type of usage. At a certain instant, the resource cost is set by considering the resource demand. Unless CSUs use the resources, they do not need to pay.

We need some more notions to tackle the resource provisioning problem in public clouds. In our work, for cost minimization, we considered reserved and spot instances, which are explained as follows.

- A flexible cost is used for *spot instances* that have a flexible utilization charge on an hourly basis.
- *Reserved instances* use hybrid cost models with fixed reservation costs. These costs vary with the contract duration and have an hourly usage charge which is lower than the charge of the spot instances.

### 1.3. Our Contributions

Our main contributions can be summarized as follows:

- To the best of our knowledge, this paper is the first work in which the uncertainty of demands and prices have been considered for the problem at hand.
- This problem is considered analytically to obtain the structure of optimal resource provisioning policies.
- A heuristic approach is proposed and shown to be near-optimal for the problem at hand.

### 1.4. Organization

The rest of this paper is organized as follows. Section 2 provides the related work. Section 3 gives the system model and problem formulation. In Section 4, a heuristic approach is presented for this resource provisioning problem. In Section 5, a policy based on this heuristic approach is proposed and shown to be near-optimal. In Section 6, numerical results show that this heuristic-based policy is efficient which is verified by the results in Section 5. Section 7 concludes this paper by providing a discussion and future directions.

## 2. Related Work

In recent years, multiple pricing schemes were investigated in [18,19,20,21,22,23,24] from different perspectives. Most of these papers considered resource provisioning problems under reserved vs. on-demand pricing schemes. Refs. [18,19] presented an analysis of various kinds of spot instances. Refs. [20,21] showed the effectiveness of multiprice schemes, such as on-demand and reservation schemes, which many CSPs follow these days. Cost optimization for a CSU was studied in [22,23,24] by considering different pricing schemes. The papers [22,23] used stochastic integer programming models to optimize the costs of SLA-aware resource provisioning in clouds. In [24], reserved and on-demand instances were considered for minimizing the total processing times for budget-limited jobs and the cost of deadline-constrained jobs. To apply these solutions, the demands for resources needed to be predicted. Based on historical data, ref. [25] developed demand-predicting models over twelve months.

Ref. [26] proposed a cloud application as solution for multiphysics/multidomain problems. For this purpose, the authors utilized cloud technologies for managing network, hardware, the operating system, and applications. In particular, related to computational demands in the resource provisioning problem, the user could have the results from any place and any device without any other concerns. The user determined the parameters of the problem, selected a more appropriate solution for the specific problem, and obtained a solution for this problem. Minimum possible resources were allocated automatically in the background without the user’s interference. Ref. [27] proposed an optimal resource allocation scheme for maximizing the utilization of available resources on a vehicular cloud which was created by vehicles. An expected average reward maximization problem was formulated as a semi-Markov decision process (SMDP) and then solved by an iterative algorithm. Numerical results showed that the proposed approach maintained the block rate as 0.2, with the priority of maximizing the utilization of available resources.

With a deterministic resource provisioning approach, many works have tackled this problem as a single-phase optimization algorithm that only considers resources with reserved contracts from IaaS providers. They do not consider the ambiguity of users’ demands. Instead, they apply deterministic provisioning schemes for future workloads under the assumption of fixed-valued demands [28,29]. Ref. [28] considered converged optical network and computing infrastructures and designed cloud service provisioning schemes for them. To address the challenge of the evaluation and exploitation of the systems working with renewable energy, stochastic linear programming (SLP) was used for proposing a new service provisioning scheme. The proposed approach achieved stability and a fast convergence to optimality. With renting cost minimization, Ref. [29] considered the scheduling problem of periodical workflow applications. The novelty of that work came from its more realistic objective function than the ones commonly considering makespan minimization. For this problem, the authors constructed an integer programming model. By considering three types of initial schedule construction methods, they developed a precedence-tree-based heuristic. Two improvement procedures were proposed based on an initial schedule. Numerical results showed that the presented policy was effective and efficient.

With a dynamic resource provisioning approach, the following papers applied elastic cloud resource provisioning mechanisms for handling the uncertainty of users’ demands. Ref. [30] constructed resource cost optimization models for periodically performed data and computationally intensive applications at hourly intervals. Ref. [31] dynamically adjusted resources to meet predicted short-term workload for cost minimization, while avoiding SLA violations. Although these approaches met varying demands better, the resultant costs became considerably larger due to the utilization of only expensive on-demand resources.

Ref. [32] proposed a framework for packing short jobs into the deals of a buying group. In this framework, flexible resource sharing was allowed among different users. Thus, it achieved resource efficiency for the provider and cost effectiveness for the cloud user. Ref. [33] proposed a cloud service framework which offered on-demand and reserved instances by considering the reservation cost-minimization problem for distributed data centers as an integer programming problem. An online rolling-horizon-based policy and an offline heuristic-greedy policy were proposed for this problem. Numerical results showed that the proposed algorithms could handle large volumes of instance demands via a higher reservation resource utilization by saving significant service costs.

Ref. [34] introduced an advanced cluster-based metaheuristic-driven energy-aware routing technique for IoT-enabled WSNs. The proposed technique aimed to achieve maximum network lifetime and energy utilization. Its performance was investigated in several aspects. Numerical results showed its enhancements over recent approaches in the literature. As a result, the suggested technique was applied for tests with a full simulation capability of NS-3.26. The simulation results showed that its performance was improved with respect to the packet delivery ratio (PDR), energy consumption, network lifetime, proportion of dead nodes, and latency.

Ref. [35] suggested a secure, cost- and energy-aware heuristic-based policy to schedule real-time workflow jobs processing IoT data by considering different security needs. That study worked with a four-tier architecture which consisted of layers of mist, IoT, fog, and cloud. Mist, fog, and cloud tiers had heterogeneous resources. The suggested technique was compared with a secure (not cost- and energy-aware) baseline strategy. Their performance was evaluated via simulations, under various security-level probabilities for the initial IoT input data of workflow jobs. Numerical results showed that the proposed technique both achieved a better QoS than the benchmark technique and reduced monetary costs by saving energy.

The paper [36] proposed techniques for determining reservation amounts, and future spot prices were not known for them. However, the first technique had an assumption of knowing future demands while the other technique made no such assumption but could ensure the reservation cost and usage of cloud resources considerably. In addition, the paper formulated the problem as an integer linear programming (ILP) problem. Numerical results demonstrated that the proposed technique achieved a considerably smaller cost than ILP.

The paper [37] presented an energy-aware cluster-based routing protocol where cluster heads (CHs) were elected via several routing metrics including distances between sink and sensors, number of neighbors, residual energy, and times when a node acts as a CH. When compared with some techniques in the literature, it was shown that the suggested technique extended the network lifetime in addition to improving throughput considerably.

In [38], the closest study to our work, reserved and on-demand instances were considered for optimizing the cost of resource reservation. First, a structure for an optimal algorithm was obtained with the knowledge of all demands during the scenario (omniscient case). Then, some low-complexity, heuristic policies were proposed and shown to be efficient.

The works for minimizing the costs mostly apply integer programming models that are naturally NP-hard. Efficient heuristics have not been found for cost optimization problems with spot instances in polynomial time. Thus, several heuristic policies have been proposed for cost optimization problems. This paper also proved that the heuristic policy achieved optimality in certain scenarios.

Table 1 provides a gap analysis in the related literature.

## 3. System Model and Problem Formulation

In this section, we first present the system model briefly. Then, we formulate the resource provisioning problem in public clouds.

### 3.1. System Model

**Figure 1:**
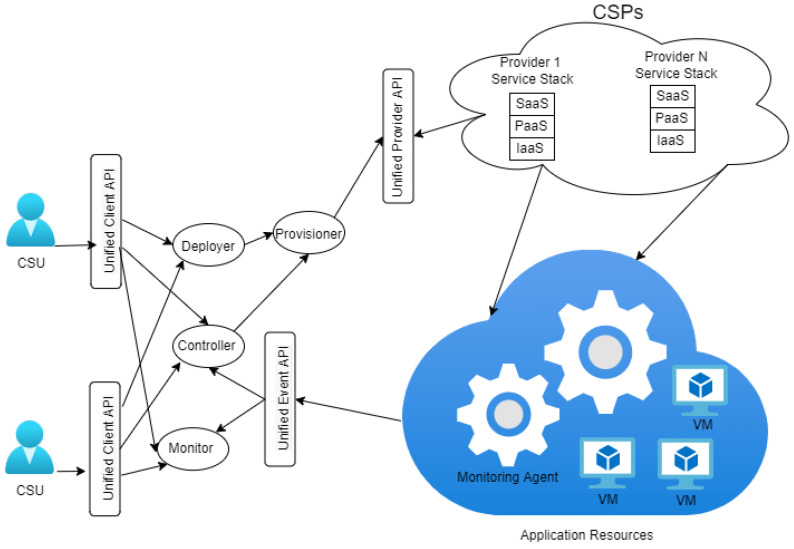
Resource provisioning framework in the public cloud.

We tackled the resource reservation problem occurring in public clouds similar to the problem in [38]. We determined the quantity of resources to be reserved for minimizing the costs of executing applications. Figure 1 shows a resource provisioning framework in public clouds.

The two main modules in this resource provisioning framework are the controller module and deployer module. The deployer module analyzes applications statically to determine their optimal resource requirement. The controller module fine-tunes the provisioned resources dynamically to alleviate underprovisioning and overprovisioning cases.

### 3.2. Problem Formulation

In this resource provisioning problem, we determined the quantity of resources to be reserved for minimizing the costs of executing an application. For the problem at hand, the following assumptions were made:

- An application is run through different stages denoted by *t*, 1≤t≤T. The number of hours per stage (*h*) determines the granularity of a stage.
- Its demand for resources, denoted by *D*, is known (or estimated using the mechanisms described in [25]) at every stage of execution of the application. The predicted values of the demand vector are available at each stage *t*, 1≤t≤T.
- Reserved resources have a one-time fixed charge for the contract duration and a variable-usage charge to be paid for hourly usage basis.
- If the demand at a given stage t is more than the reserved resource, that difference between the demanded and reserved instances is made up with spot instances.

A CSP offers *K* different types of reservation contract. Each type of contract (*k*) is associated with a one-time reservation cost ($R_{k}$), a usage cost ($r_{k}$) per hour, and its duration ($t_{k}$) in number of stages. At every stage (*t*), the number of instances to be reserved ($x_{t}^{R_{k}}$) needs to be decided. We also need to determine the number of instances to be launched based on the reservation from contract *k* ($x_{t}^{r_{k}}$) in addition to spot instances ($x_{t}^{o_{k}^{j}}$). The cost of spot instances is more than the usage cost of reserved instances ($o_{k}^{j}>r_{k}$). Meanwhile, a reservation increase incurs high reservation costs. Therefore, by balancing these two factors, we aimed to find an optimal reservation.

$Cost_{t}$ denotes the cost at any given stage *t*, i.e,

(1) $$Cost_{t}≜\sum_{k=1}^{K}\left(x_{t}^{R_{k}}R_{k}+x_{t}^{r_{k}}r_{k}h+x_{t}^{o_{k}^{j}}o_{k}^{j}h\right).$$

The first, second, and last terms in (1) stand for the reservation cost under contract *k*, the usage costs of the reserved instances, and the costs of using spot instances, respectively. There exist some policies which can minimize the cost in (1) optimally. However, there is no polynomial-time policy for an optimal solution of the problem since an integer programming problem is NP-hard. Therefore, we looked for low-complexity algorithms to solve the problem at hand.

## 4. Heuristic-Based Resource Reservation

Here, some heuristic policies are derived for the resource provisioning problem in polynomial times. It is shown that when there is a single-type contract *k* with a contract duration of $t_{k}$ stages, and the demand vector is available for stages $t=1,\ldots ,t_{k}$, it is possible to determine the optimal value for the reservation under contract *k*. It is assumed each stage lasts 1 h. The usage cost for a demand *d* in a single stage with *x* reserved instances under contract *k* is:

(2) $$\begin{array}{ccc} Cost_{u}\left(x,d\right) & ≜ & \left\{\begin{array}{cc} d\cdot r_{k} & \mathrm{if}d\leq x\\ x\cdot r_{k}+\left(d-x\right)\cdot o & \mathrm{if}d>x \end{array}\right. \end{array}$$

(3) $$\begin{array}{ccc} & \;\;\;\;\;\;\;\;= & r_{k}\cdot min\left(x,d\right)+o\cdot max\left(d-x,0\right) \end{array}$$

### Heuristic with Known Demand Vector

**Figure 2:**
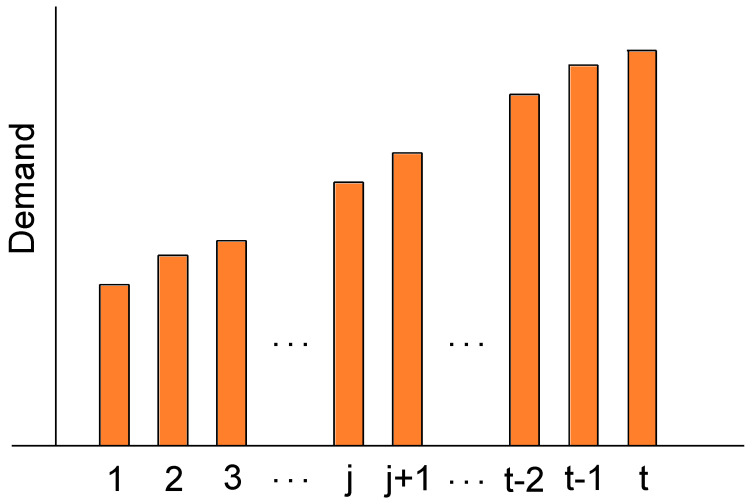
Sorting demands in a contract of duration *t*.

In this subsection, an approach similar to that in [38] is used to sort the demands in a single contract duration. Please see Figure 2 for an example of sorting demands in a contract of duration $t_{k}$.

It is assumed that a vector of demands *D* for the duration of $t_{k}$ stages is available. *x* denotes the quantity of resources reserved under contract *k*; $E_{x}$ denotes the total cost corresponding to demand vector *D*, which consists of reservation costs and resource-usage costs. Hence, by combining Equation (1) with reservation costs, $E_{x}$ is

(4) $$E_{x}=x\cdot R_{k}+\sum_{i,D_{i}\leq x}D_{i}\cdot r_{k}+\sum_{i,D_{i}>x}\left[x\cdot r_{k}+\left(D_{i}-x\right)\cdot o_{D_{i}}\right]$$

where the spot price, denoted by $o_{D_{i}}$, is defined for the demand in hour *i*, $D_{i}$, as

(5) $$o_{D_{i}}≜r_{k}+\alpha_{k}\cdot D_{i},$$

where $\alpha_{k}$ is a positive constant determined by contract *k*.

As an example for spot pricing in the previous Equation (5), let us consider the following example.

**Figure 3:**
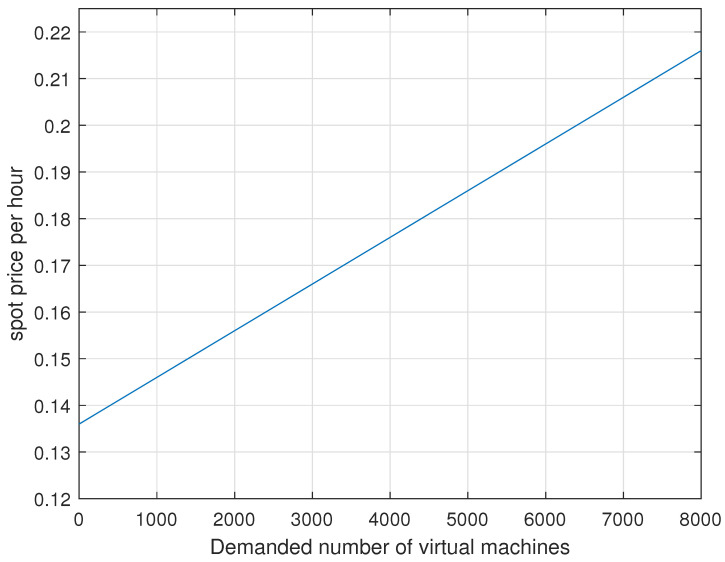
Spot prices per hour vs. the number of demanded virtual machines (VMs) with the hourly usage cost as 0.136 USD/h and the α parameter as 0.00001.

**Example** **1.** *For this example, let us take the hourly usage cost as 0.136 USD/h and the α parameter as 0.00001. Let us consider the number of demanded virtual machines (VMs) from 0 to 8000 with a mean of 4000. Please see Figure 3 for the trend of spot prices per hour vs. the number of demanded virtual machines (VMs). Under this spot pricing, the hourly usage cost becomes 0.216 USD/h for a demand of 8000 VMs whereas the hourly usage cost becomes 0.136 USD/h for no demanded VM.*

From (5), the spot price can be expressed for the demand in hour *j*, $D_{j}^{s}$, as

(6) $$\begin{array}{ccc} o_{k}^{j} & = & r_{k}+\alpha_{k}D_{j}^{s},\\ & = & r_{k}+a_{k}^{j} \end{array}$$

If the number of reserved instances per hour is set to the demand level $D_{j}^{s}$, the cost can be expressed as

(7) $$E_{D_{j}^{s}}=D_{j}^{s}\cdot R_{k}+\sum_{i=1}^{j}D_{i}^{s}\cdot r_{k}+\sum_{i=j+1}^{t_{k}}\left[D_{i}^{s}\cdot r_{k}+\left(D_{i}^{s}-D_{j}^{s}\right)\cdot o_{k}^{i}\right].$$

From (6), (7) yields

(8) $$\begin{array}{ccc} E_{D_{j}^{s}} & = & D_{j}^{s}\cdot R_{k}+\sum_{i=1}^{j}D_{i}^{s}\cdot r_{k}+\sum_{i=j+1}^{t_{k}}\left[D_{i}^{s}\cdot r_{k}+\left(D_{i}^{s}-D_{j}^{s}\right)\cdot \left(r_{k}+a_{k}^{i}\right)\right]\\ & = & D_{j}^{s}\cdot R_{k}+\sum_{i=1}^{t_{k}}D_{i}^{s}\cdot r_{k}+\sum_{i=j+1}^{t_{k}}\left(D_{i}^{s}-D_{j}^{s}\right)\cdot a_{k}^{i}. \end{array}$$

Similarly, if the number of reserved instances per hour is set to the demand level $D_{j+1}^{s}$, the cost can be expressed as

(9) $$E_{D_{j+1}^{s}}=D_{j+1}^{s}\cdot R_{k}+\sum_{i=1}^{t_{k}}D_{i}^{s}\cdot r_{k}+\sum_{i=j+2}^{t_{k}}\left(D_{i}^{s}-D_{j+1}^{s}\right)\cdot a_{k}^{i}.$$

From (8) and (9), the cost difference is

(10) $$\begin{array}{ccc} \Delta E_{D_{j}^{s},D_{j+1}^{s}}\;\; & = & \;\;\left(D_{j+1}^{s}-D_{j}^{s}\right)\cdot R_{k}+\sum_{i=j+2}^{t_{k}}\left(D_{i}^{s}-D_{j+1}^{s}\right)\cdot a_{k}^{i}-\sum_{i=j+1}^{t_{k}}\left(D_{i}^{s}-D_{j}^{s}\right)\cdot a_{k}^{i}\\ \;\; & = & \;\;\left(D_{j+1}^{s}-D_{j}^{s}\right)\cdot R_{k}+\sum_{i=j+2}^{t_{k}}\left(D_{i}^{s}-D_{j+1}^{s}-D_{i}^{s}+D_{j}^{s}\right)\cdot a_{k}^{i}-\left(D_{j+1}^{s}-D_{j}^{s}\right)\cdot a_{k}^{j+1}\\ \;\; & = & \;\;\left(D_{j+1}^{s}-D_{j}^{s}\right)\cdot \left(R_{k}-\sum_{i=j+1}^{t_{k}}a_{k}^{i}\right) \end{array}$$

By using (10), we obtain the structure of optimal policy. First, we provide the following lemmas.

**Lemma** **1.** *$R_{k}<\sum_{i=1}^{t_{k}}a_{k}^{i}$ for all contract type k.*

**Proof.**  The proof is by contradiction. Assume that $R_{k}\geq \sum_{i=1}^{t_{k}}a_{k}^{i}$. Then, from (10), $\Delta E_{D_{0}^{s},D_{1}^{s}}\geq 0$ (note that $E_{D_{0}^{s}}=0$) and $\Delta E_{D_{j}^{s},D_{j+1}^{s}}>0$ for $1\leq j\leq t_{k}$. This implies that all increases in the level of reserved instances increase the total cost instead of decreasing it. In this case, it is better not to reserve any instance. Therefore, for any contract type *k*, the following inequality should hold for a CSU to reserve an instance from the CSP, $R_{k}<\sum_{i=1}^{t_{k}}a_{k}^{i}$.    □

**Lemma** **2** ([38], Lemma 2). *The number of instances to be reserved, for which the total cost is minimized, is always a member of the demand vector.*

**Theorem** **1.** *For a single type contract k, with demand vector D available for $t=1,2,\ldots t_{k}$ stages, there exists a value of*

$$j_{k}=\arg\min_{j}\left|R_{k}-\sum_{i=j+1}^{t_{k}}a_{k}^{i}\right|,$$

*such that the cost is minimum with the reservation of $D_{j_{k}}^{s}$.*

**Proof.**  From (6) and (10),

(11) $$\frac{\Delta E_{D_{j}^{s},D_{j+1}^{s}}}{D_{j+1}^{s}-D_{j}^{s}}=R_{k}-\sum_{i=j+1}^{t_{k}}\alpha_{k}\cdot D_{i}^{s}.$$

From Lemma 1,

(12) $$R_{k}<\sum_{i=1}^{t_{k}}\alpha_{k}\cdot D_{i}^{s}.$$

Therefore,

(13) $$R_{k}-\sum_{i=j+1}^{t_{k}}\alpha_{k}\cdot D_{i}^{s}<0$$

for some *j* and

(14) $$R_{k}-\sum_{i=j+1}^{t_{k}}\alpha_{k}\cdot D_{i}^{s}>0$$

for other *j*.

Since $D_{j+1}^{s}-D_{j}^{s}\geq 0$$\forall 1\leq j\leq t_{k}$,

$$\begin{array}{ccc} E_{D_{1}^{s}} & \geq & E_{D_{2}^{s}}\geq \ldots \geq E_{D_{j_{k}}^{s}}\\ E_{D_{j_{k}}^{s}} & \leq & E_{D_{j_{k}+1}^{s}}\leq \ldots \leq E_{D_{t_{k}}^{s}} \end{array}$$

where

(15) $$\begin{array}{ccc} j_{k} & ≜ & \arg\min_{j}\left|R_{k}-\sum_{i=j+1}^{t_{k}}a_{k}^{i}\right|\\ & = & \arg\min_{j}\left|R_{k}-\alpha \sum_{i=j+1}^{t_{k}}D_{i}^{s}\right|. \end{array}$$

Hence, if the reservation is made for a quantity of resources equal to $D_{j_{k}}^{s}$, the cost is minimized from Equation (15).    □

## 5. Heuristic Resource Reservation Policies

In the previous section, we derived heuristic-based resource reservation schemes analytically. By benefiting from these derivations in the previous section, we look for robust heuristic policies in this section.

In this section, we propose three heuristic policies for this problem: single-contract resource reservation policy, no-resource reservation policy, mean-resource reservation policy.

### 5.1. Single-Contract Resource Reservation Policy (SCRRP)

A single-contract resource reservation policy is proposed by the intuition from the heuristic approach in the previous section. Just one contract (*k*) is considered with the SCRRP. Contract *k* is denoted as $\left(R_{k};t_{k},a_{k}\right)$ where $R_{k}$ is the reservation cost, $t_{k}$ is the contract duration (in stages), and $a_{k}$ is the discount on the usage cost over a spot resource.

In this policy, first, the demand vector $D\left[1,\ldots ,T\right]$ is sorted as in previous section. Then, we look for

$$j_{k}=\arg\min_{j}\left|R_{k}-\alpha \sum_{i=j+1}^{t_{k}}D_{i}^{s}\right|$$

under contract *k*. See Algorithm 1.
**Algorithm 1** Single-Contract Resource Reservation Policy**Input:** Demand vector $D\left[1,\ldots ,T\right]$, contract *k*.   **(1)** $D^{s}\leftarrow sort\left(D\right)$;   **(2)** $lastsum≜\frac{R_{k}}{\alpha }$ **from Theorem 1;**      i=0**;**      sum=0**;**      **while** (sum<lastsum) **do**         i=i+1**;**         $sum=sum+D^{s}\left(T-i+1\right)$**;**      **endwhile**   **(3) Number of reserved instances** ←$D^{s}\left(T-i+1\right)\times T$**;**

### 5.2. Mean-Resource Reservation Policy (MRRP)

With mean-resource reservation policy, a CSU decides to reserve the average/mean of the hour-based demanded instances in one day (the first day of the contract) during a contract duration of 1 year. See Algorithm 2. Although this policy is smarter than the no-resource reservation policy, it cannot reduce the cost as much as the single-contract resource reservation policy. This can be used as another benchmark policy for the comparison with the single-contract resource reservation policy.
**Algorithm 2** Mean-Resource Reservation Policy**Input:** Demand cector $D\left[1,\ldots ,T\right]$, Contract *k*.   **(1)** $D^{s}\leftarrow sort\left(D\right)$**;**      **# The mean of the hour-based demands in one day is reserved for each hour in a year.**   **(2) Number of reserved instances** ←mean(D(1:24))×T**;**

### 5.3. No-Resource Reservation Policy (NRRP)

With the no-resource reservation policy, a CSU decides not to reserve any instance during the contract duration. In this case, the CSU has to pay the spot price determined by the CSP at each hour. Therefore, the policy is not smart, but it can show how much a smart policy can make a difference.

## 6. Numerical Results

The heuristic-based resource reservation policies (SCRRP, MRRP, NRRP) were applied. Then, the performance values of the policies were compared with each other in terms of the total cost and cost percentage with respect to the no-resource reservation policy. For this purpose, the reserved pricing models Amazon EC2 offers were taken. A one-year contract was considered for the reserved pricing model. The demand vector of VMs was formed with an exponential distribution via mean = 4000 VMs and a uniform distribution with mean = 4000 VMs. From the Amazon EC2 pricing model, the reservation cost (one-year) was considered as USD 243 and the reserved VM (one-month) usage cost was considered as 0.136 USD/h. Moreover, we chose α=0.00001 to determine the spot price from (5) (we chose α=0.00002 in the last subsection).

Our mean-resource reservation policy (MRRP) was almost the same structurally as the realistic reservation and scheduling with spot price (RRS-SP) in [36]. In fact, the MRRP was better because it considered 12-month average of demands while the RRS-SP considered just a 1-month average. Therefore, we did not compare our policies with the RRS-SP.

In this section, we investigate the performance of heuristic-based resource reservation policies for the following three cases in three subsections. In Section 6.1, this paper investigates exponentially distributed demand traffic with α=0.00001. In Section 6.2, this paper investigates Poisson distributed demand traffic with α=0.00001. In Section 6.3, this paper investigates Poisson distributed demand traffic with α=0.00002. Hence, we consider differences in both the distributions and values of the α parameter.

### 6.1. Exponential Demand Traffic

**Figure 4:**
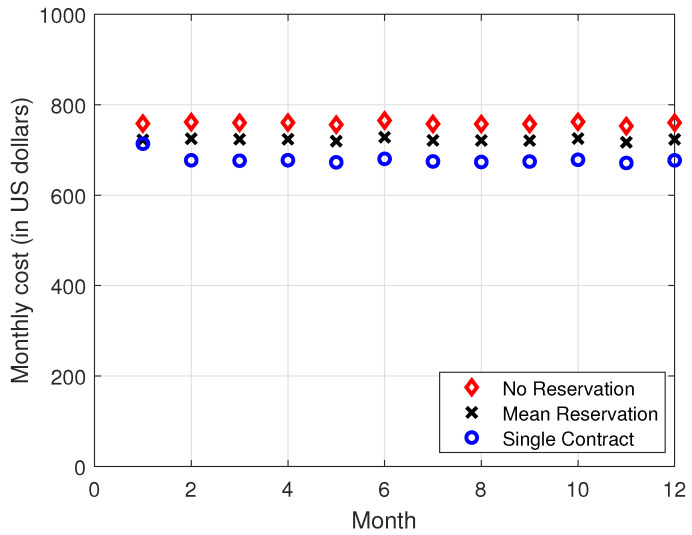
Monthly cost vs. month. The demand traffic is modeled as an exponential stochastic process with mean = 4000 VMs. Monthly cost (in USD).

**Figure 5:**
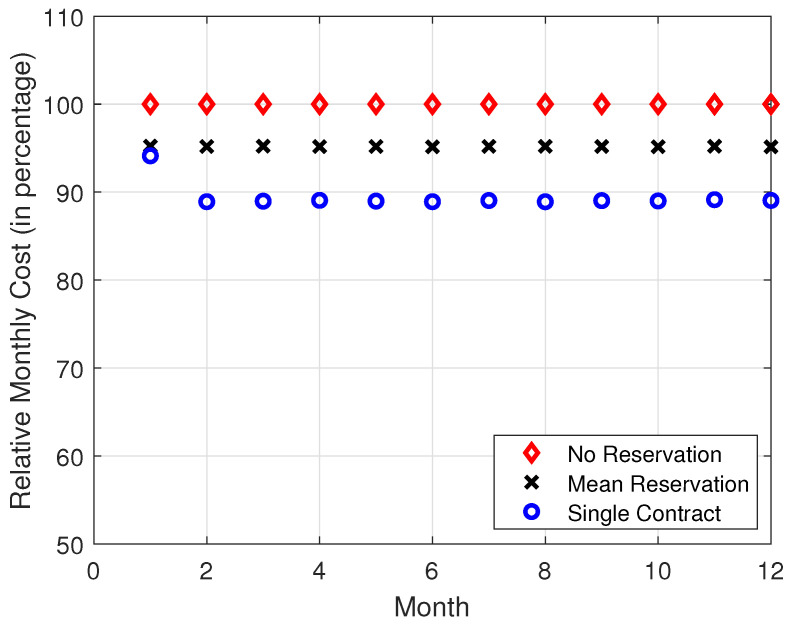
Relative monthly cost vs. month. The demand traffic is modeled as an exponential stochastic process with mean = 4000 VMs. Monthly cost (in USD).

**Figure 6:**
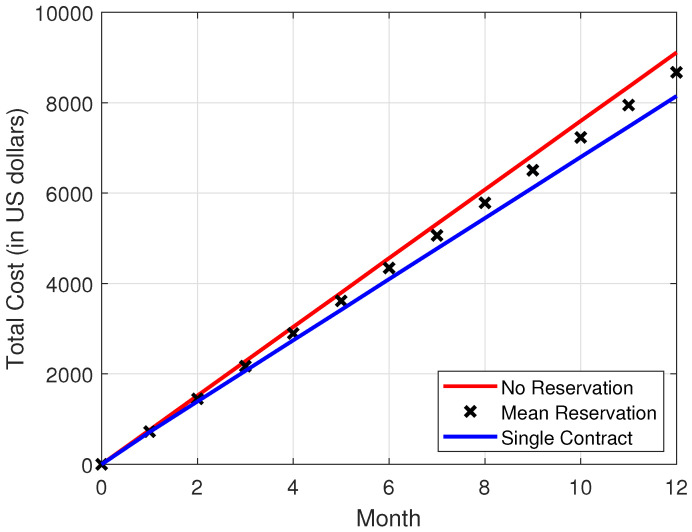
Total cost vs. month. The demand traffic is modeled as an exponential stochastic process with mean = 4000 VMs. Monthly cost (in USD).

**Figure 7:**
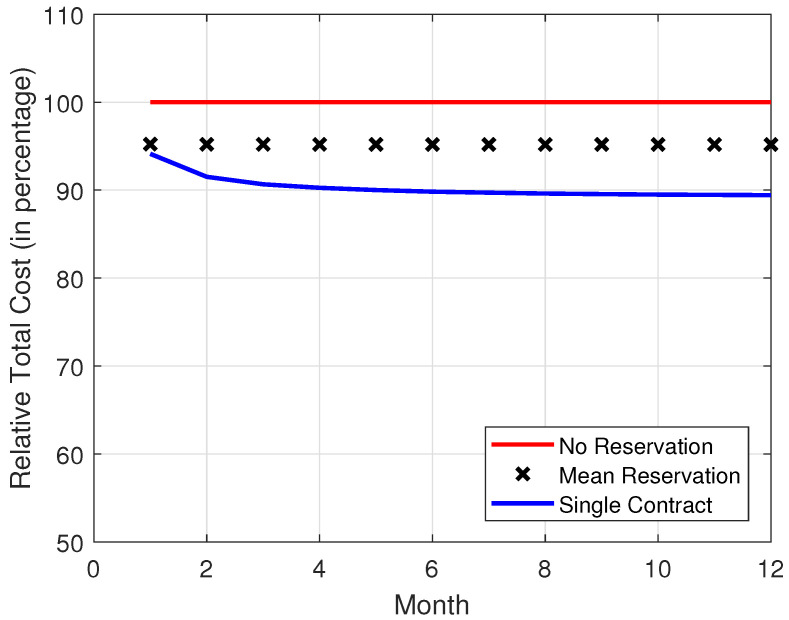
Relative total cost vs. month. The demand traffic is modeled as an exponential stochastic process with mean = 4000 VMs. Monthly cost (in USD).

In Figure 4, Figure 5, Figure 6 and Figure 7, it is observed that the SCRRP shows the best performance compared with the MRRP and NRRP. In other words, the SCRRP has the least cost among the three policies. Moreover, the cost under the MRRP is much less than under the NRRP.

In Figure 4, we see that the NRRP achieves a monthly cost of USD 760. In Figure 5, we see that the SCRRP’s total cost is considerably less than NRRP. In fact, it reduces the monthly cost more than 11% compared with that of the NRRP under exponential traffic. Moreover, the MRRP achieves a 4.8% lower monthly cost than the NRRP, both under exponential demand traffic.

In Figure 6, we see that the NRRP achieves an annual cost of USD 9110. In Figure 7, the SCRRP reduces the annual cost by more than 11% compared with that of the NRRP under exponential traffic. Moreover, the MRRP achieves a 4.8% lower annual cost than the NRRP, both under exponential demand traffic.

Regarding Figure 4, Figure 5, Figure 6 and Figure 7, we wish to make a remark. The relative performance of different resource reservation policies was affected slightly by the distribution of the demand data because we performed 10,000 Monte Carlo trials.

### 6.2. Poisson Demand Traffic

**Figure 8:**
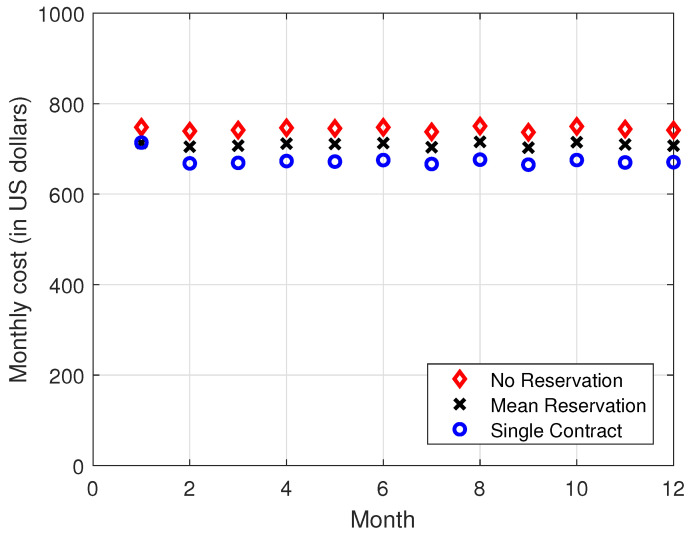
Monthly cost vs. month. The demand traffic is modeled as a Poisson stochastic process with mean = 4000 VMs. Monthly cost (in USD).

**Figure 9:**
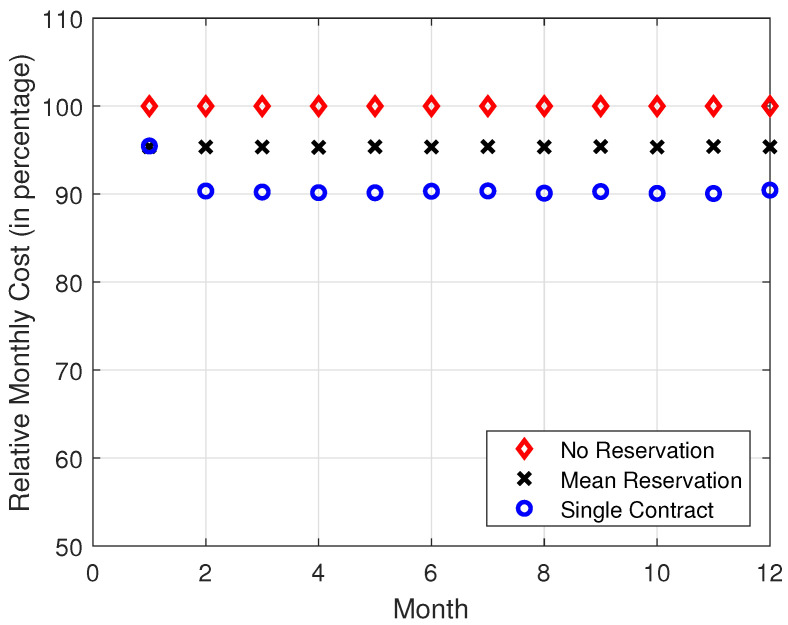
Relative monthly cost vs. month. The demand traffic is modeled as a Poisson stochastic process with mean = 4000 VMs. Monthly cost (in USD).

**Figure 10:**
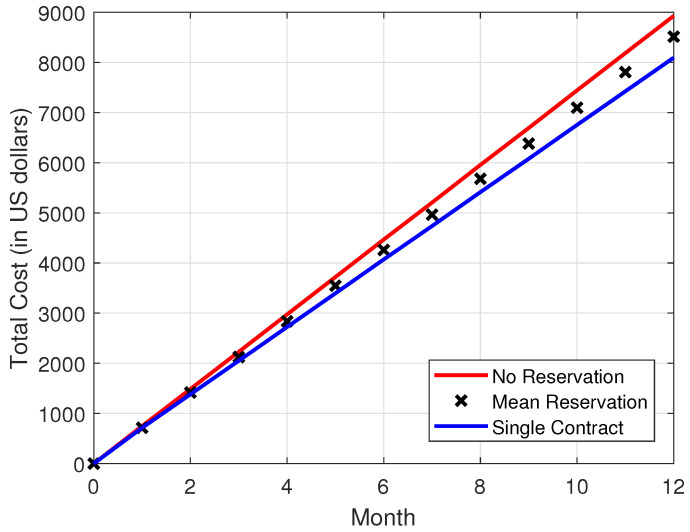
Total cost vs. month. The demand traffic is modeled as a Poisson stochastic process with mean = 4000 VMs. Monthly cost (in USD).

**Figure 11:**
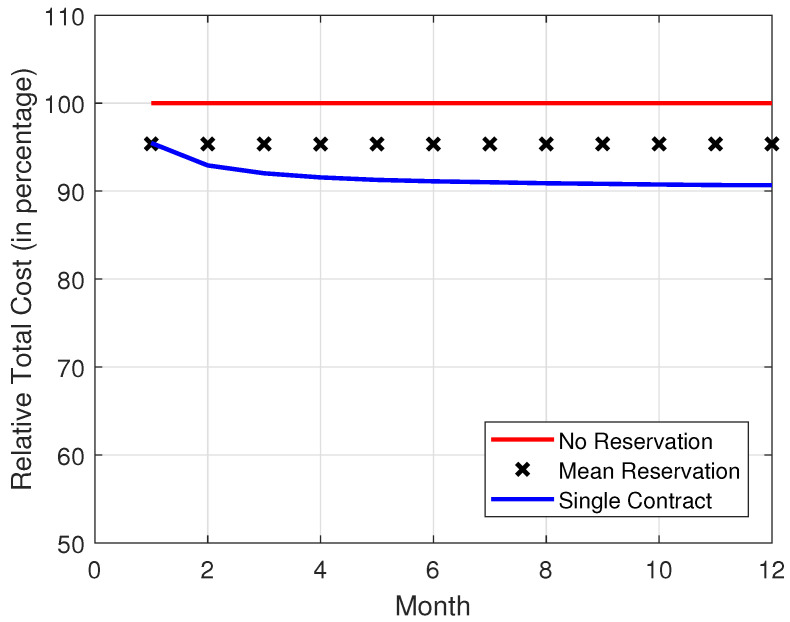
Relative total cost vs. month. The demand traffic is modeled as a Poisson stochastic process with mean = 4000 VMs. Monthly cost (in USD).

In Figure 8, Figure 9, Figure 10 and Figure 11, it is observed that the SCRRP shows the best performance compared with the MRRP and NRRP. In other words, the SCRRP has the least cost among the three policies. Moreover, the cost under the MRRP is much less than that under the NRRP.

In Figure 8, we see that the NRRP achieves a monthly cost of USD 750. In Figure 9, we see that the SCRRP makes the total cost considerably less than the NRRP. In fact, it reduces the monthly cost more than 9.5% compared with the NRRP under Poisson traffic. Moreover, the MRRP achieves a 4.6% lower monthly cost than the NRRP, both under Poisson demand traffic.

In Figure 10, we see that the NRRP achieves an annual cost of nearly USD 9000. In Figure 11, the SCRRP reduces the annual cost by more than 9.33% compared with the NRRP under Poisson traffic. Moreover, the MRRP achieves a 4.63% lower annual cost than the NRRP under Poisson demand traffic.

By considering Figure 4, Figure 5, Figure 6, Figure 7, Figure 8, Figure 9, Figure 10 and Figure 11, we wish to make a remark. The relative performance of different resource reservation policies were affected slightly from the distribution of the demand data. Because we performed 10,000 Monte Carlo trials, the differences caused by the distributions are smoothed.

### 6.3. Poisson Demand Traffic with Larger Alpha Values

From Table 2, it is observed that the SCRRP shows the best performance compared with the MRRP and NRRP. In other words, the SCRRP has the least cost among the three policies. Moreover, the cost under the MRRP is much less than that under the NRRP.

From Table 2, we see that the NRRP achieves a monthly cost of USD 944 whereas the SCRRP provides a total cost of USD 648, considerably less than the NRRP. In fact, it reduces the monthly cost by more than 30.8% compared with the no-resource reservation policy under exponential traffic. Moreover, the MRRP achieves USD 763, a 19.2% lower monthly cost than the NRRP, both under exponential demand traffic.

From Table 2, the SCRRP reduces the annual cost to USD 7808, nearly 31.0% less than the NRRP, which has a total cost of USD 11,319 under Poisson traffic. Moreover, the MRRP achieves an annual cost of USD 9141, 19.2% less than the NRRP under Poisson demand traffic.

## 7. Conclusions and Future Works

### 7.1. Conclusions

This work investigated resource reservation problems occurring in public clouds. First, the problem was investigated analytically, and the structure of the optimal policy was derived. Then, we proposed a heuristic policy, the single-contract reservation policy, to solve the cloud resource provisioning problem in polynomial time. It was analytically proved that the single-contract resource reservation policy became efficient under a pricing scheme with reserved and spot instances. In addition, the mean-resource reservation policy was proposed as a simpler heuristic which performed better than the no-resource reservation policy, although it could not reduce the cost as much as the single-contract resource reservation policy. The polynomial-time heuristics enabled us to work on hourly demand data for a duration of 1 year or more with no difficulties. It is concluded that the proposed heuristic policy makes the total cost considerably less than the no-resource reservation policy.

### 7.2. Discussion and Future Works

In this work, we considered a spot pricing scheme. On the other hand, different spot pricing schemes can be used depending on the cloud service providers. In this work, we generated demand traffic, but datasets for cloud workloads could also be used. In this paper, we considered CPU bounds, but there exist some other requirements such as IO bounds.

As future work, we plan to work on achieving optimality or near-optimality in the case where CSUs do not know the demand vector. In addition, different spot pricing schemes can be considered. In our future work, we also plan to work with datasets for cloud workloads instead of generating demand traffic. As other future work, we can consider the different types of instances and requirements. The novel concepts and approaches in this paper can give insight to those scholars who investigate the problem with similar pricing schemes in the beyond 5G and IoT era.

## Figures and Tables

**Table 1:** Brief comparison of resource provisioning policies in public clouds.

Related Works	On-Demand Pricing	Spot Pricing	Knowing Demands	Not Knowing Demands
[18]	no	yes	yes	no
[19]	no	yes	yes	no
[20]	yes	no	yes	no
[21]	yes	no	yes	no
[22]	no	yes	yes	no
[23]	no	yes	yes	no
[24]	yes	no	yes	no
[32]	yes	no	yes	no
[33]	yes	no	yes	yes
[36]	no	yes	yes	yes
[38]	yes	no	yes	yes
This work	no	yes	yes	yes

**Table 2:** Monthly cost with Poisson demand traffic with α=0.00002.

Month	1	2	3	4	5	6	7	8	9	10	11	12
NRRP	937	951	944	938	947	949	934	941	945	937	952	944
MRRP	756	767	763	758	765	766	754	761	763	756	769	763
SCRRP	685	651	648	645	650	651	641	646	648	644	651	648

## Data Availability

Not applicable.

## Abbreviations

The following abbreviations are used in this manuscript:

5G	fifth-generation (communications)
IoT	Internet of things
UAV	uncrewed aerial vehicle
SLA	service level agreement
VM	virtual machine
CSU	cloud service user
CSP	cloud service provider
SMDP	semi-Markov decision process
SLP	stochastic linear programming
NP	nonpolynomial
NRRP	no resource reservation policy
MRRP	mean resource reservation policy
SCRRP	single-contract resource reservation policy
